# The phosphorylation status of PIP5K1C at serine 448 can be predictive for invasive ductal carcinoma of the breast

**DOI:** 10.18632/oncotarget.26357

**Published:** 2018-11-20

**Authors:** Nisha Durand, Sahra Borges, Tavia Hall, Ligia Bastea, Heike Döppler, Brandy H. Edenfield, E. Aubrey Thompson, Xochiquetzal Geiger, Peter Storz

**Affiliations:** ^1^ Department of Cancer Biology, Mayo Clinic Comprehensive Cancer Center, Mayo Clinic, Jacksonville, FL 32224, USA; ^2^ Laboratory Medicine and Pathology, Mayo Clinic, Jacksonville, FL 32224, USA

**Keywords:** PIP5K1C, breast cancer, invasive phenotype, phosphorylation

## Abstract

Phosphatidylinositol-4-phosphate 5-kinase type-1C (PIP5K1C) is a lipid kinase that regulates focal adhesion dynamics and cell attachment through site-specific formation of phosphatidylinositol-4,5-bisphosphate (PI4,5P_2_). By comparing normal breast tissue to carcinoma *in situ* and invasive ductal carcinoma subtypes, we here show that the phosphorylation status of PIP5K1C at serine residue 448 (S448) can be predictive for breast cancer progression to an aggressive phenotype, while PIP5K1C expression levels are not indicative for this event. PIP5K1C phosphorylation at S448 is downregulated in invasive ductal carcinoma, and similarly, the expression levels of PKD1, the kinase that phosphorylates PIP5K1C at this site, are decreased. Overall, since PKD1 is a negative regulator of cell migration and invasion in breast cancer, the phosphorylation status of this residue may serve as an indicator of aggressiveness of breast tumors.

## INTRODUCTION

The family of phosphatidylinositol-4-phosphate 5-kinase type-1 (PIP5K1) lipid kinases consists of three isoforms, PIP5K1A, PIP5K1B and PIP5K1C, each of which exists in multiple alternatively spliced variants. PIP5K1 enzymes regulate formation of PI4,5P_2_ at distinct locations within cells, using phosphatidylinositol-4-phosphate (PI4P) as a substrate [1]. However, little is known with respect to the roles of these enzymes in cancer development or progression. In breast cancer it was shown that *PIP5K1A* gene copy numbers, together with other genes such as *AKT3*, *PI4KB* and *PI3KC2B* in an amplification stretch in chromosome 1q, are increased in a large percentage of tumors [2]. In glioblastoma multiforme, copy number amplifications in chromosome 19 have been described leading to increased expression of *PIP5K1C*, *AKT2* and *PIK3R2* [2]. In addition, differential expression of *PIP5K1B*, *PIP5K1C* and *PIP4K2B* has been described for lung adenocarcinoma [3]. The regulation of these gene clusters suggests altered phosphoinositide lipid signaling and lipid-regulated trafficking in these cancers.

While little is known on the cellular functions of the two other PIP5K1 enzymes, PIP5K1C (PIP5K1γ) has been shown to be a major regulator of focal adhesion (FA) dynamics [4]. Depletion of PIP5K1C leads to cytoskeletal changes and severe attachment defects in cells [5]. Altered FA dynamics due to decrease in PIP5K1C activity or expression has been linked to increased cell migration and invasion [6, 7]. PIP5K1C localization to FA is negatively-regulated by p35/Cdk5-mediated phosphorylation at S650 [8]; and PIP5K1C degradation is regulated by phosphorylation through p70S6K1 at threonine 553 and serine 555 [7], while its lipid kinase activity is inhibited after phosphorylation through protein kinase D (PKD) at serine 448 [9].

Members of the PKD family of serine/threonine kinases control multiple functions within cells by phosphorylating a broad spectrum of targets [10]. In breast cancer all three isoforms (PKD1, PKD2 and PKD3) have been implicated in regulating cancer cell survival and proliferation during tumor formation [11–14]. However, with respect to cell migration and invasiveness, it was shown that PKD1 blocks these events through multiple mechanisms. These include PKD1-induced changes in the stability of cell-cell contacts [15–17], in focal adhesion dynamics [9, 17], in actin reorganization dynamics [18–20] and in filopodia formation and stabilization [21]. Additionally, PKD1 has been shown to block epithelial-to-mesenchymal transition (EMT) [22–24], and to mediate changes in the expression of matrix metalloproteinases (MMPs) [25]. Consequently, in breast cancer, the transition from a less aggressive to a metastatic phenotype is characterized by *PRKD1* (PKD1) gene promoter methylation and downregulation of PKD1 expression [14, 26].

We here investigated if expression of PIP5K1C or its phosphorylation status at serine 448 can be indicative for invasive breast cancers. Our data suggest that PKD1 expression levels in tumors correlate with PIP5K1C phosphorylation at serine 448, and that the PIP5K1C phosphorylation at this residue may be a predictive marker for progression to an aggressive phenotype.

## RESULTS

### The expression level of PIP5K1C is not predictive for breast cancer survival or subtype

**Figure 1:**
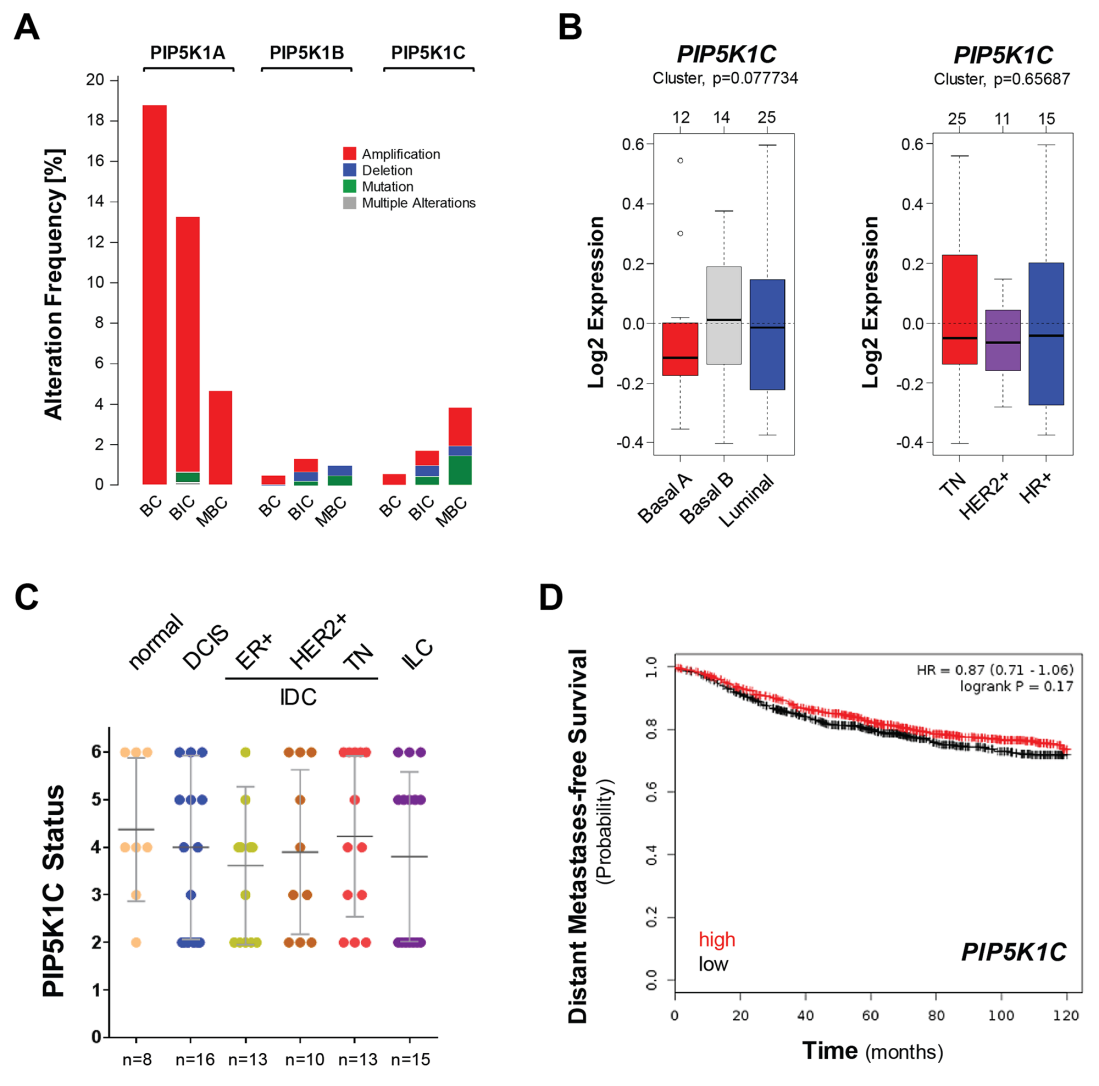
The expression of *PIP5K1C* is not predictive for breast cancer survival or subtype **(A)** Percent alteration frequency (mutations or alterations in expression) of *PIP5K1A, PIP5K1B and PIP5K1C* in 3 studies: breast cancer (BC; n = 2509 samples; [42]), breast invasive carcinoma (BIC; n = 1105 samples; TCGA) and mutational profiles of metastatic breast cancers (MBC; n=216 samples; [43]). The analysis was performed using cBioPortal (http://www.cbioportal.org/public-portal/index.do). **(B)** Relative expression of *PIP5K1C* in breast cancer cell lines (n=51) grouped into basal or luminal subtypes (left side) or grouped into TN, HER2+ or HR+ subtypes (right side). The analysis was performed using GOBO from Lund University (http://co.bmc.lu.se/gobo/). **(C)** Tissue microarrays with indicated groups of samples were immunohistochemically-stained for PIP5K1C expression. Relative expression was determined and rated from 0-6 (0 = no expression; 6 = strongest expression). **(D)** Distant metastases-free survival (DMFS) of breast cancer patients with high or low expression of *PIP5K1C* over time. The analysis was performed with the Kaplan-Meier Plotter (http://kmplot.com/analysis/index.php?p=service&cancer=breast) using standard settings. Patient samples (n=1746) were split by median, the follow up threshold was set 10.

Alterations in PIP5K1C expression or activity have been linked to increased cell migration and invasion [6, 7]. We used cBioPortal (http://www.cbioportal.org/public-portal/index.do) to analyze three different available datasets, including 2509 breast cancer (BC) samples, 1105 invasive breast carcinoma (IBC, BIC) samples, or 216 metastatic breast cancers (MBC) for gene alterations such as amplification, deletion or mutational events in *PIP5K1A*, *PIP5K1B* and *PIP5K1C* genes. While we detected gene amplification of *PIP5K1A*, which is consistent with previously published data [2], in all three datasets, the alteration frequency for PIP5K1B and PIP5K1C was very low (Figure 1A).

However, the *PIP5K1C* gene amplification frequency slightly increased when comparing the BC to MBC datasets which prompted us to further determine if the expression levels of *PIP5K1C* can be predictive for breast cancer subtypes or aggressiveness. Therefore, we first investigated a panel of 51 breast cancer cell lines using GOBO from Lund University (http://co.bmc.lu.se/gobo/) (Figure 1B). Cells were grouped either into basal or luminal subtypes (left side) or grouped into TN, HER2+ or HR+ subtypes (right side). We did not observe a statistical difference in *PIP5K1C* expression within these groups.

Next, we tested if PIP5K1C protein expression is altered during progression of breast cancer. Therefore, we analyzed progression tissue microarrays (TMAs) including normal breast tissue, ductal carcinoma *in situ* (DCIS), 3 groups of invasive ductal carcinomas (ER positive; HER2 positive; or TN) as well as invasive lobular carcinoma (ILC) using IHC. Our results suggest that levels of total PIP5K1C are not indicative for BC progression (Figure 1C). Eventually, we determined if *PIP5K1C* gene expression levels can be linked to distant metastases free survival (DMFS) in patients. Therefore, we analyzed a set of 1746 patient samples for which gene expression data was available. Samples were split by median and DMFS plotted over time. The analysis was performed using the Kaplan-Meier Plotter (http://kmplot.com/analysis/index.php?p=service&cancer=breast), previously described [27]. We did not detect a statistical difference between patients with high or low expression of PIP5K1C with respect to DMFS (Figure 1D).

Overall, our data lead to the conclusion that PIP5K1C expression levels are not significantly changed in different subtypes of breast cancer and also are not indicative for patient survival.

### Phosphorylation of PIP5K1C at S448 is decreased in invasive ductal carcinoma of the breast

**Figure 2:**
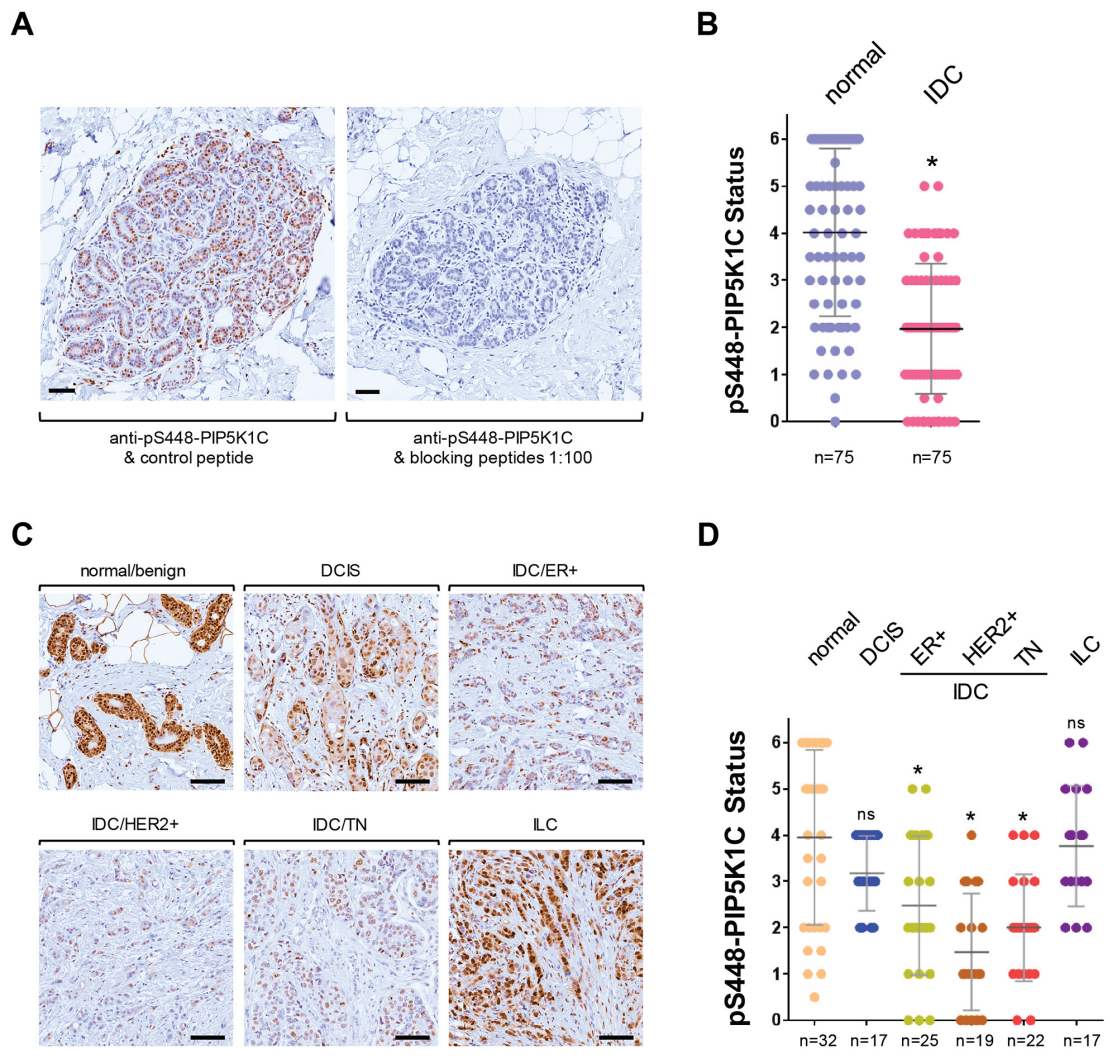
Phosphorylation of PIP5K1C at S448 is decreased in invasive ductal carcinoma of the breast **(A)** Antibody specificity control. Immunohistochemical staining of normal breast tissue. Samples were stained for pS448-PIP5K1C alone or in the presence of blocking phospho-peptides (at 1:100) to demonstrate antibody specificity for this serine phosphorylation site. The bar indicates 100 μm. **(B)** Indicated groups of samples were immunohistochemically-stained for pS448-PIP5K1C. Relative expression was determined and rated from 0-6 (0 = no expression; 6 = strongest expression). The asterisk indicates statistical significance n < 0.0001, when compared to normal tissue. **(C)** Representative pictures of pS448-PIP5K1C expression in normal/benign breast tissue, DCIS, ILC and different IDC subgroups. The bar indicates 100 μm. **(D)** Tissue microarrays with indicated groups of samples were immunohistochemically-stained for pS448-PIP5K1C. Relative expression was determined and rated from 0-6 (0 = no expression; 6 = strongest expression). The asterisk indicates statistical significance when compared to normal tissue; ns = not significant as compared to normal tissue.

We previously have shown that PIP5K1C lipid kinase activity is inhibited after phosphorylation at serine 448, and have generated a phospho-specific antibody (pS448-PIP5K1C) for this site [9]. Therefore, we investigated if the phosphorylation status of PIP5K1C at S448 is altered with increasing invasiveness of breast cancers. First, we confirmed specificity of our pS448-PIP5K1C antibody by using phosphorylated blocking peptides on normal breast tissue (Figure 2A). Using this antibody we determined the phosphorylation status in a set of n = 75 samples of IDC or normal breast tissue, and found a significant decrease in PIP5K1C phosphorylation at S448 in IDC (Figure 2B). To further dissect this into IDC subgroups, we then analyzed our progression tissue microarrays (TMAs) including normal breast tissue, ductal carcinoma *in situ* (DCIS), 3 groups of invasive ductal carcinomas (ER positive; HER2 positive; or TN) as well as invasive lobular carcinoma (ILC) for this phosphorylation. Quantification of the pS448-PIP5K1C phosphorylation status after immunohistochemical (DAB staining) analysis indicated a statistically significant decrease in different subtypes of invasive ductal carcinoma of the breast, but not in ILC (representative pictures in Figure 2C, quantification in Figure 2D).

### Phosphorylation of PIP5K1C at S448 in breast cancer cells is mainly mediated by PKD1

**Figure 3:**
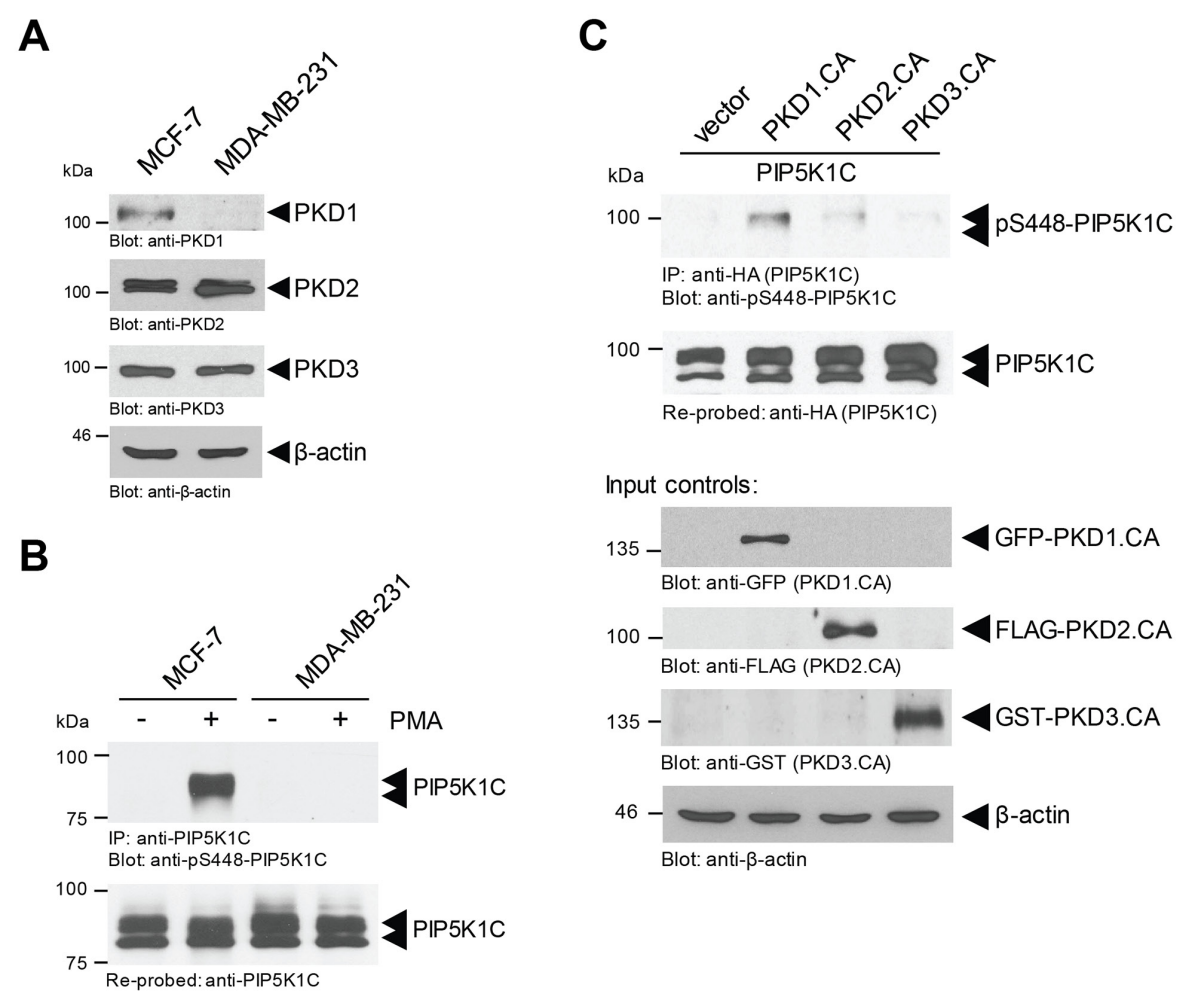
Phosphorylation of PIP5K1C at S448 in BC cells is mediated by PKD1 **(A)** Indicated cell lines were analyzed by SDS-PAGE and immunoblotting for endogenous expression of PKD1 (anti-PKD1), PKD2 (anti-PKD2) or PKD3 (anti-PKD3). Immunoblotting with anti-β-actin served as a loading control. **(B)** Indicated cell lines were treated with DMSO control or PMA (100 nM) for 10 min. Cells were lysed, endogenous PIP5K1C was immunoprecipitated (anti-PIP5K1C), and immunoprecipitates were analyzed by SDS-PAGE and immunoblotting for phosphorylation of PIP5K1C at S448 (anti-pS448-PIP5K1C). Samples were re-probed for total PIP5K1C. **(C)** MCF-7 cells were transfected with tagged constitutively-active versions of PKD1, PKD2 or PKD3 together with HA-tagged PIP5K1C. Cells were lysed, overexpressed PIP5K1C was immunoprecipitated (anti-HA), and immunoprecipitates were analyzed by SDS-PAGE and immunoblotting for phosphorylation of PIP5K1C at S448 (anti-pS448-PIP5K1C). Samples were re-probed for total PIP5K1C by staining with anti-HA. In addition expression of active PKD isoforms was determined by Western blotting of lysates with TAG-specific antibodies as indicated.

We previously have shown that in Hek293T cells the phosphorylation of PIP5K1C at S448 can be mediated by PKD1 and PKD2 enzymes [9]. In order to determine which of these two PKD isoforms are responsible for PIP5K1C phosphorylation in breast cancer cells, we compared MCF-7 cells, which express all three PKD isoforms, to MDA-MB-231 cells, which only express PKD2 and PKD3 (Figure 3A). After stimulation of PKD activity with PMA, we found that PIP5K1C is phosphorylated at S448 in MCF-7 cells, but not in MDA-MB-231 cells (Figure 3B). This result was confirmed with a second cell line (SK-BR-3) that also lacks PKD1 expression (Supplementary Figure 1A). As a negative control we also determined the phosphorylation status at S650 (Supplementary Figure 1B), which in contrast to S448 is negative-regulatory and is not within a PKD phosphorylation motif [8].

Above data suggest that PKD1 may be the PKD isoform that is responsible for PIP5K1C phosphorylation at S448. To test this we determined the phosphorylation status of PIP5K1C at S448 and S650 (negative control) after expression of constitutively-active versions of PKD1, PKD2 and PKD3 in MCF-7 cells. Our data indicate that PKD1 in breast cancer cells indeed is the main regulator of PIP5K1C phosphorylation at S448 (Figure 3C), but not at S650 (control, Supplementary Figure 1C).

### The PKD1 expression status indicates invasive ductal carcinoma

**Figure 4:**
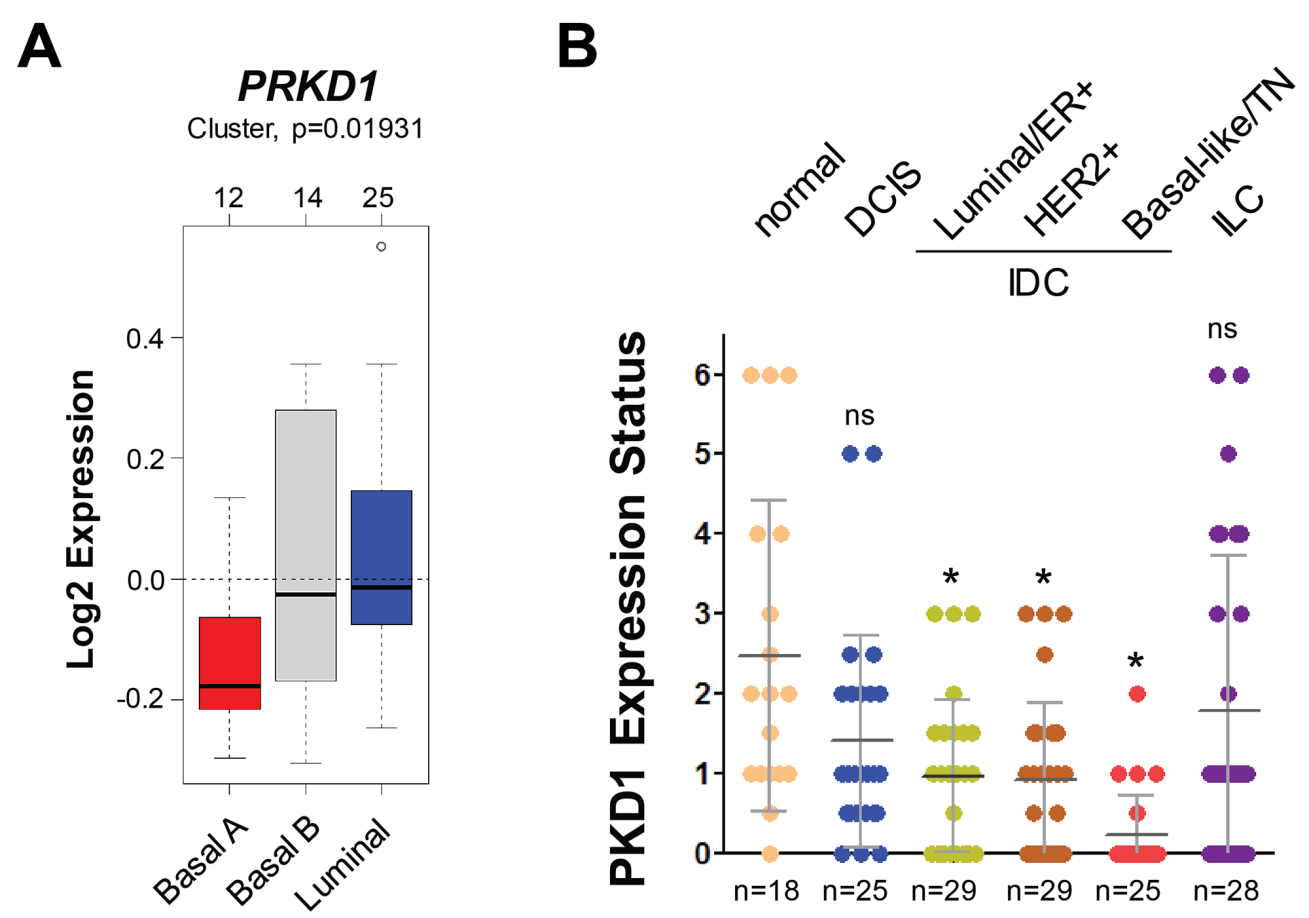
The PKD1 expression status indicates invasive ductal carcinoma **(A)** Relative expression of *PRKD1* (PKD1) in breast cancer cell lines (n=51) grouped into basal or luminal subtypes. The analysis was performed using GOBO from Lund University (http://co.bmc.lu.se/gobo/). **(B)** Tissue microarrays with indicated groups of samples were immunohistochemically-stained for PKD1. Relative expression was determined and rated from 0-6 (0 = no expression; 6 = strongest expression). The asterisk indicates statistical significance when compared to normal tissue; ns = not significant as compared to normal tissue.

PKD1 expression previously has been shown to be downregulated in invasive breast carcinoma through epigenetic silencing of its *PRKD1* gene promoter [26]. A comparison between 51 luminal and basal subtypes of breast cancer cell lines using GOBO from Lund University (http://co.bmc.lu.se/gobo/) [28] suggested a further (statistically significant) decrease in *PRKD1* gene expression in breast cancers of the basal A type (Figure 4A). Moreover, the immunohistochemical (DAB staining) analysis of progression tissue microarrays (TMAs) including normal breast tissue, ductal carcinoma *in situ* (DCIS), 3 groups of invasive ductal carcinomas (ER positive; HER2 positive; or TN) indicated that levels of total PKD1 are significantly decreased in invasive ductal carcinoma (Figure 4B), correlating with the data obtained for pS448-PIP5K1C in (Figure 2D).

### PKD1 regulates the phosphorylation of PIP5K1C *in vivo* in orthotopic tumors

**Figure 5:**
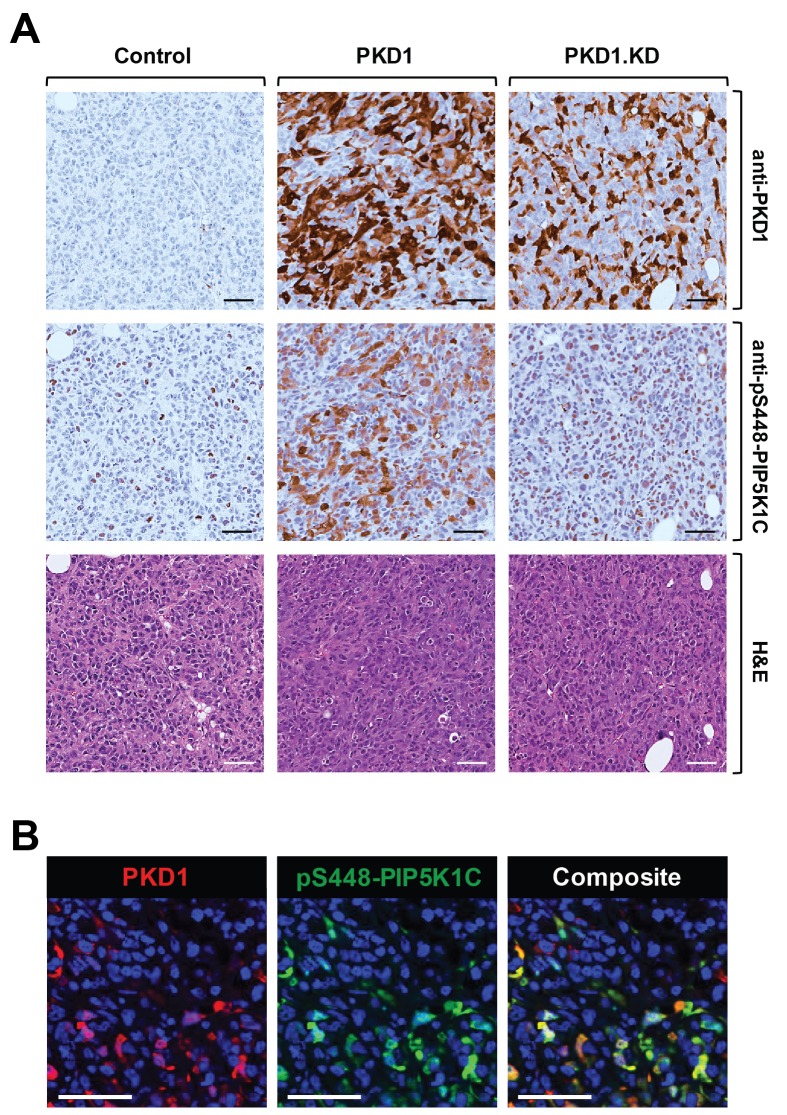
PKD1 regulates phosphorylation of PIP5K1C at S448 in orthotopic tumors *in vivo* **(A, B)** Analyses of 5 week old primary tumors of MDA-MB-231 cells stably expressing vector control, wildtype PKD1 or a kinase-dead version (PKD1.KW) orthotopically-injected into the mfp of mice (experiment described in [29]). (A) Shown are IHC analyses of a representative tumor area for PKD1 (anti-PKD1 antibody) and for PIP5K1C phosphorylated at S448 (anti-pS448-PIP5K1C) as well as H&E staining. (B) Shown is an immunofluorescence-IHC analyses of a representative tumor area for co-occurrence of PKD1 expression (red; anti-PKD1 antibody) and PIP5K1C phosphorylated at S448 (green; anti-pS448-PIP5K1C) in tumor cells. In A and B the scale bar indicates 50 μm.

We next analyzed previously-generated mouse orthotopic tumors in which we had implanted MDA-MB-231 cells into the mammary fat pad (mfp) of nude mice [29]. MDA-MB-231 cells are highly invasive and do not express PKD1 due to epigenetic downregulation of its promoter [26]. We previously had shown that ectopic expression of PKD1 in these cells led to a decrease in their invasiveness and decreased tumor burden, and that this is dependent on PKD1 kinase activity, since the expression of a kinase-dead variant of PKD1 did not show differences to the vector control [29]. We utilized these available tumor samples to determine if PKD1 regulates phosphorylation of PIP5K1C *in vivo*. As predicted, tumors formed by control-transfected cells did not show PKD1 expression or significant phosphorylation of PIP5K1C at S448 (Figure 5A). However, tumors generated with cells, in which PKD was ectopically re-expressed, also showed PIP5K1C phosphorylation at S448. On the other hand, introduction of a kinase-dead version of PKD1 (PKD1.KD) did not alter the PIP5K1C phosphorylation status at S448 in tumors.

Since IHC were performed on serial sections, in order to demonstrate that presence of PKD1 and phosphorylation of PIP5K1C at S448 occur in the same tumor cells, we performed co-immunofluorescence analyses on our tissues. We found that cells that express high levels of PKD1 also expressed high levels of pS448-PIP5K1C (Figure 5B), further supporting our findings that PKD1 regulates the phosphorylation of PIP5K1C *in vivo*.

### PKD1 expression status and PIP5K1C phosphorylation correlate in patient samples

**Figure 6:**
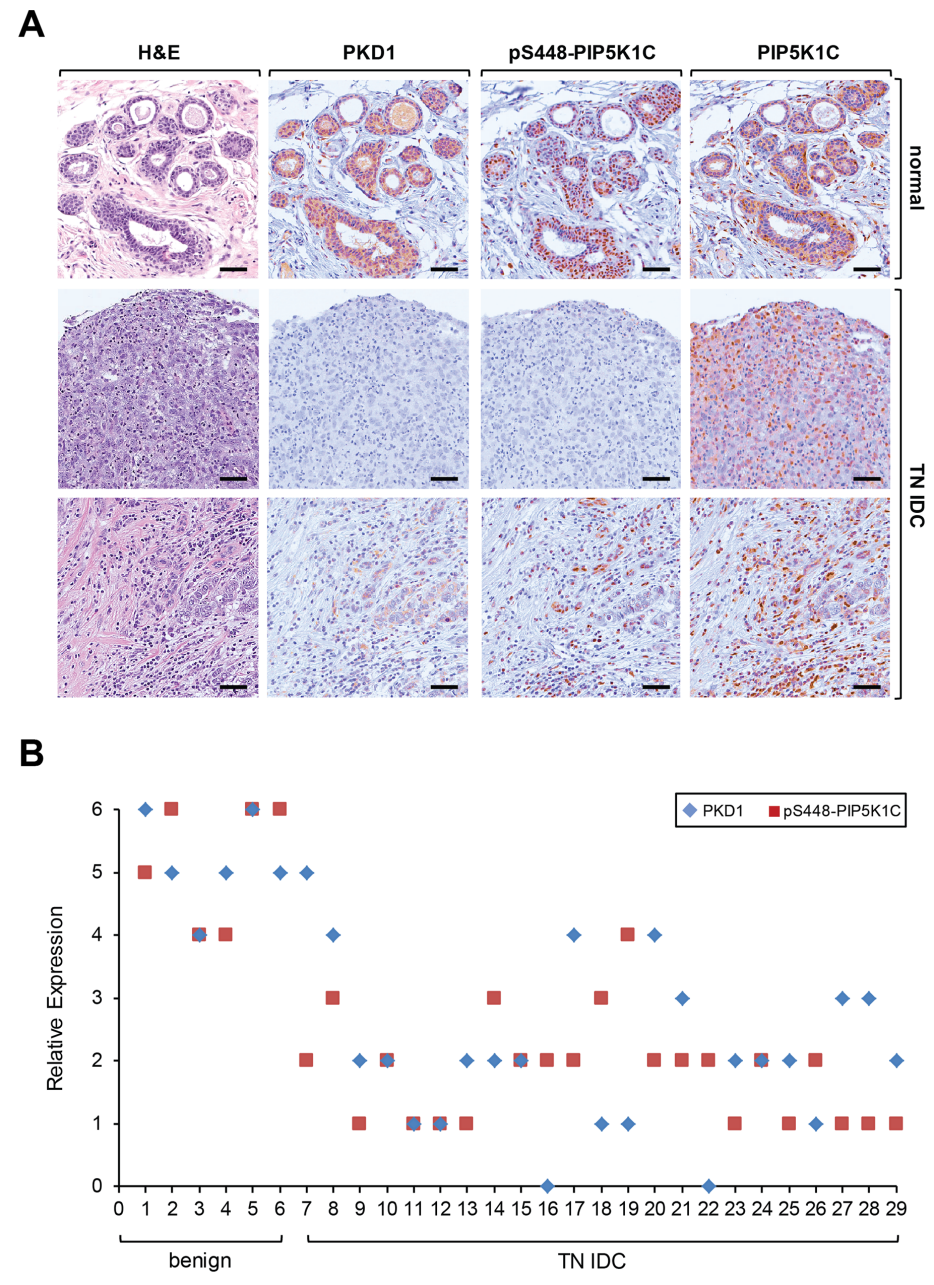
PKD1 expression status and phosphorylation of PIP5K1C at S448 correlate in patient samples of TNBC **(A)** Relative expression of PKD1, pS448-PIP5K1C and PIP5K1C in patient samples. Shown are a representative normal sample and two different representative IDC patient samples stained by H&E or by IHC using anti-PKD1, anti-pS448-PIP5K1C and anti-PIP5K1C antibodies. **(B)** Relative expression of PKD1 and pS448-PIP5K1C in benign and TN IDC. The quantification analysis is described in Materials and Methods.

We next determined if there is a direct correlation between PKD1 expression and PIP5K1C phosphorylation at S448 in invasive ductal carcinoma. As previously published [25, 26] we found PKD1 abundantly expressed in normal tissue, but downregulated in IDC. Moreover, presence of PKD1 correlated with PIP5K1C phosphorylation at S448, whereas the overall levels of PIP5K1C expression were comparable to normal controls (Figure 6A). This prompted us to perform a more detailed analysis of an increased number of samples. A direct comparison of patient tissue of benign tissue cases and TNBC samples showed a correlation between PKD1 expression and PIP5K1C phosphorylation at S448 (Figure 6B). A Spearman’s Rho calculation indicated a correlation coefficient of R = 0.48107 and the two-tailed value of p = 0.00825. By normal standards, this association between the two variables (PKD1 and pS448 expression) is considered statistically significant. Additionally, a Metagene Score analysis indicated statistical significance (p < 0.001) between benign and TNBC groups (Supplementary Figure 2).

Taken together, our data suggests that the phosphorylation status of PIP5K1C at serine 448 can be predictive for invasive ductal carcinoma of the breast.

## DISCUSSION

Alterations in activity of phosphoinositide kinases and associated changes in phosphoinositide signaling are important events driving breast cancer formation and progression. PI4,5P_2_ is the most abundant phosphoinositide and regulates a multitude of cellular processes at the plasma membrane and at other organelle membranes [30]. The majority of PI4,5P_2_ in cells is produced by PIP5K1 lipid kinases, which phosphorylate PI4P as a substrate [1]. PI4,5P_2_ can function as a substrate for phosphatidylinositol 3-kinase (PI3K) or for phospholipase C (PLC), but also serve as an adapter lipid to promote recruitment of proteins to the plasma membrane. In such a function it regulates many aspects of vesicular transport and actin dynamics [31].

In cancer cells it was shown that PIP5K1C-induced synthesis of PI4,5P_2_ at the leading edge of cells drives the formation of membrane protrusion and facilitates directional cell migration [32]. In addition to this, oncogenic, activating mutations in the catalytic subunit of PI3K, the kinase that demands PI4,5P_2_ as a substrate, have been detected in numerous breast cancer subtypes 25-40% [33]. Based on these findings we investigated if PIP5K1C expression levels are altered in invasive breast cancers. However, our analyses suggested that gene amplification of *PIP5K1C* is not significant in breast cancer, whereas amplifications of *PIP5K1A* were detected (Figure 1A). This is in congruence with published findings showing that a large percentage of breast cancers have amplification of chromosome 1q genes, including *PIP5K1A* [2]. Moreover, the expression status of PIP5K1C was unchanged in breast cancer patient samples and cell lines (Figures 1B, 1C), and PIP5K1C expression seems not to be indicative of distant metastases-free survival (Figure 1D).

Downregulation of lipid kinase activity due to phosphorylation events has been shown for different PIP5K1 enzymes. For example, PIP5K1B is phosphorylated and inactivated by AMPK and PKC [34]. Similarly a negative regulation by phosphorylation through PKD1 at S448 has been shown for PIP5K1C [9]. Using a previously characterized [9] phospho-specific antibody directed against this site, we here show that phosphorylation of PIP5K1C at S448 can be indicative for invasive breast cancer (Figure 2).

Besides inhibiting PIP5K1C activity through phosphorylation [9], PKD1 also targets other phosphatidylinositol lipid kinases such as type II PIP kinase [35] to affect PI4,5P_2_ levels. Moreover, PKD1 has been shown to associate with type II PI4K and type I PI4,5K [36]; and to regulate the lipid kinase activity of PI4KIIIβ to modulate PI4P levels [37]. This suggests PKD enzymes as regulators of phosphoinositide signaling at multiple levels and locations within cells.

Previous *in vitro* and *in vivo* work indicated that PKD1, if expressed in breast cancer, prevents cell migration and invasion at multiple levels [11, 38, 39]. On the other hand, unlike PKD1, PKD2 and PKD3 have been shown to increase cell motility and invasion [11, 40, 41]. Consequently, the transition from a less aggressive to a metastatic phenotype is characterized by *PRKD1* (PKD1) gene promoter methylation and downregulation, and an upregulation PKD2 and PKD3 [14, 26]. As expected, due to their function in regulating cell migration, out of the three PKD isoforms only PKD1 is a significant regulator of PIP5K1C phosphorylation (and activity) in breast cancer cells (Figure 3; and [9]).

In IDC patient samples, we found a significant downregulation of PKD1 expression (Figure 4B). Moreover, PKD1 expression levels/activity and PIP5K1C phosphorylation at S448 are functionally linked in breast cancer, as shown by analyses of orthotopically-implanted tumors in an animal model (Figure 5). Analyses of patient samples suggest that in invasive breast cancer the downregulation of PKD1 correlates with a decrease of PIP5K1C phosphorylation at S448 (Figure 6).

Overall our data indicate that while PIP5K1C expression levels cannot be used as predictive marker for type or outcome in breast cancer, the phosphorylation status at S448 of this lipid kinase correlates with aggressiveness. A decrease in this phosphorylation event is observed in all IDC either from ER+, HER2+ or TN cancers. Since phosphorylation of this site is mediated by PKD1, a kinase that previously was linked to maintain the epithelial phenotype and decrease of migratory potential of cancer cells, we predict that the phosphorylation status of this residue may serve as an indicator of aggressiveness of breast tumors. However, future studies are needed to determine if it also could be an indicator of treatment response towards a less migratory phenotype.

## MATERIALS AND METHODS

### Ethics statement

This investigation has been conducted in accordance with the ethical standards according to the Declaration of Helsinki and in accordance to national and international guidelines and has been approved by the Mayo Clinic Institutional Review Board (IRB).

### Cell lines

MCF-7, MDA-MB-231 and SK-BR-3 cells were obtained from ATCC (ATCC, Manassas, VA, USA). All cell lines have been verified by Gene Print 10 STR profiling (Genetic Resources Core Facility at John’s Hopkins University School of Medicine; latest verifications occurred between June and October, 2018) and have been routinely tested for mycoplasma (IDEXX Bioresearch, Columbia, MO, USA). MCF-7 and MDA-MB-231 cells were maintained in DMEM with 10% FBS; and SK-BR-3 in McCoy’s 5a Modified Medium with 10% FBS.

### Antibodies, reagents and DNA constructs

All antibodies used for immunoprecipitation, immunoblotting, immunofluorescence or immunohisto-chemistry are described in detail in Table 1. The phosphorylation-specific anti-pS650-PIP5K1C antibody was a gift from Dr. De Camilli and is described in [8]. The anti-pS448-PIP5K1C antibody was made by 21^st^ Century Biochemicals (Marlboro, MA, USA) and is further described in [9]. Ac-NTVFRKN[pS]SLKSSPSK-Ahx-C-amide and C-Ahx-SNTVFRKN[pS]SLKSSPS-amide were used as blocking peptides for this antibody. Secondary HRP-linked antibodies were from Millipore (Billerica, MA, USA) and secondary antibodies for immunofluorescence (Alexa Fluor 488 F(ab’)2 fragment of goat-anti-rabbit IgG or Alexa Fluor 568 F(ab’)2 fragment of goat-anti-mouse) were from Invitrogen (Grand Island, NY). 12-Phorbol 13-myristate acetate (PMA) was from Sigma (St. Lois, MO, USA). Expression plasmids for HA-tagged PIP5K1C, as well as the expression constructs for tagged constitutively-active versions (S to E mutations in critical activation loop serines) of PKD1, PKD2 or PKD3 have been described in detail elsewhere [9]. Lipofectamine 2000 (Invitrogen) was used for transient transfections.

**Table 1:** Sources, dilutions and concentrations of antibodies used

Antibody	Company/Source	Catalog Number	Species	IHC	IF/IF-IHC	WB	IP
PIP5K1C/PIP5K1γ	Millipore	ABS190	rabbit	1:250			
PIP5K1C/PIP5K1γ (MAO-R1)	Abcam	ab109192	rabbit			1:1000	2 µl/sample
pS448-PIP5K1C/PIP5K1γ	Storz Laboratory	N/A	rabbit	1:1000	1:1000	1:500	
pS650-PIP5K1C/PIP5K1γ	De Camilli Laboratory	N/A	rabbit			1:500	
PKD1	Storz Laboratory	N/A	mouse	1:400	1:2000	1:1000	
HA (12CA5)	Sigma-Aldrich	11583816001	mouse			1:5000	1 µl/sample
GST (Z5)	Santa Cruz	sc-459	rabbit			1:2000	
PKD2	Millipore	07-488	rabbit			1:2000	
β-actin	Sigma-Aldrich	A5441	mouse			1:2000	
GFP (B2)	Santa Cruz	sc-9996	mouse			1:2000	
FLAG (M2)	Sigma-Aldrich	F3165	mouse			1:2000	
PKD3	Bethyl Laboratories	A300-319A	rabbit			1:2000	

### Immunoblotting, immunoprecipitation and SDS-PAGE

Cells were washed twice with cold (4°C) PBS (140 mM NaCl, 2.7 mM KCl, 8 mM Na_2_HPO_4_, 1.5 mM KH_2_PO_4_, pH 7.2). After lysis with lysis buffer (50 mM Tris-HCl pH7.4, 1% Triton X-100, 150 mM NaCl, 5 mM EDTA pH 7.4) plus Protease Inhibitor Cocktail (PIC, Sigma-Aldrich), samples were incubated on ice (30 min), centrifuged at 13,000 rpm (15 min, 4 °C) and protein concentration was determined. As indicated, lysates then were analyzed by Western blot or subjected to immunoprecipitation. For immunoprecipitation, lysates were incubated with 2 μg of target-specific antibody for one hour, followed by incubation with protein G-Sepharose beads (GE Healthcare, Piscataway, NJ, USA) for 30 minutes. Immune-complexes were washed 3 times with TBS (50 mM Tris-HCl pH 7.4, 150 mM NaCl), and then resolved in 20 μl TBS and 2x Laemmli buffer. Samples were subjected to SDS-PAGE, transferred to nitrocellulose membranes and visualized by immunostaining.

### Tissue microarrays (TMAs)

Tissue samples were initially collected with the approval of the Mayo Clinic Institutional Review Board. Benign (“normal”) tissue samples were either from reduction mammoplasty or benign surgical cases. For the breast cancer samples, the variables were if tumors were ductal or lobular (no special tumor types were included), then if ductal tumors were invasive or *in-situ* (DCIS), and then whether the IDC was ER+, HER2+, or TN. No other variables such as laterality, grade, age, LVI or nodal status, or size of tumor were considered. Generation and analyses of the TMAs was performed under protocol 09-000530. Therefore, all unique patient identifiers and confidential data were removed and tissue samples were de-identified. All data was analyzed anonymously. For Figure 2B, we incorporated analysis of an additional TMA (BRN801b) from US Biomax (Rockville, MD) to increase sample numbers for normal tissue (adjacent normal or hyperplasia of mammary glands).

### Orthotopic animal model

The animal experiment in Figure 5 was performed under protocols (A15207 and A14810) approved by the Mayo Clinic Institutional Animal Care and Use Committee (IACUC), and data on tumor growth has been published elsewhere [29]. In short, seven mice (female, nu/nu) per experimental group were orthotopically-injected with MDA-MB-231 cell lines stably-expressing PKD1 wildtype, a kinase-dead (KD) version of PKD1 (PKD1.K612W) or control plasmid (for details see [29]). At week 5 (end point) primary tumors were removed, evaluated (see [29]), fixated with formalin and embedded in paraffin for further immunohistochemical analysis.

### Immunohistochemistry and immunofluorescence on tissues

Slides were de-paraffinized (xylene, three times for 5 min each), rehydrated with ethanol (100%, 95%, 75%, twice with each concentration for 3 min each), rinsed in water and subjected to antigen retrieval as described by the manufacturer (Agilent, Santa Clara, CA, USA). After antigen retrieval in 10 mM sodium citrate buffer (pH 6.0), slides were treated with 3% H_2_O_2_ (5 min), washed with PBS containing 0.5% Tween 20, and blocked with protein block serum-free solution (Agilent) for 5 min at room temperature (RT). For immunohistochemistry, anti-PIP5K1C, anti-pS448-PIP5K1C or anti-PKD1 antibodies were diluted in Antibody Diluent Background Reducing Solution (Agilent) and visualized using the EnVision Plus Anti-Rabbit Labelled Polymer Kit (Agilent). Images were scanned using the ScanScope XT scanner and ImageScope software (Aperio, Vista, CA, USA). For immunofluorescence, blocked sections were incubated with anti-pS448-PIP5K1C or anti-PKD1 antibodies in Antibody Diluent Background Reducing solution (Agilent) at 4 °C, overnight. After 3 washes with PBS plus 0.05 % Tween-20, Alexa Fluor 488- or Alexa Fluor 568-labeled secondary antibodies were added at a 1:500 dilution (RT, 1 hr). Eventually, DAPI (final concentration 125 μg/ml) was added for 15 min. LabVision PermaFluor (Thermo Scientific) was used as mounting medium. Images were captured by a fluorescent scanner (ScanScope FL, Aperio) and processed using ImageScope software (Aperio).

### Analysis of TMAs

The TMAs were scored independently by three different experienced scientists. Uniform pre-established criteria were used. Immunoreactivity was graded semiquantitatively by considering the intensity of the staining of the ductal cells. A histological score was obtained from each sample, which ranged from 0 (no immunoreaction) to 6 (maximum immunoreactivity). Reproducibility of the scoring method between three observers was greater than 90%. In the remaining cases, in which discrepancies had been noted, differences were settled by consensus review of corresponding slides.

### Statistical analysis

Data are presented as mean ± SD. P values were acquired with the student’s *t*-test using Graph Pad software, and p < 0.05 was considered statistically significant.

## SUPPLEMENTARY MATERIALS FIGURES



## Abbreviations

BC: breast cancer
BIC: breast invasive carcinoma
DCIS: ductal carcinoma *in situ*
DMFS: distant metastases-free survival
ER: estrogen receptor
HER: human epidermal growth factor receptor 2
HR: hormone receptor
IDC: invasive ductal carcinoma
ILC: invasive lobular carcinoma
MBC: metastatic breast cancer
PIP5K: phosphatidylinositol-4-phosphate 5-kinase
PKD: protein kinase D
TN: triple-negative
