# Supplementary material for: The phosphorylation status of PIP5K1C at serine 448 can be predictive for invasive ductal carcinoma of the breast
Source: Oncotarget. 2018 Nov 20;9(91):36358–70. doi: 10.18632/oncotarget.26357 (PMC6284740; doi:10.18632/oncotarget.26357)
Supplement: Supplementary file 1 [file oncotarget-09-36358-s001.pdf]

The phosphorylation status of PIP5K1C at serine 448 can be predictive for invasive ductal carcinoma of the breast

SUPPLEMENTARY MATERIALS

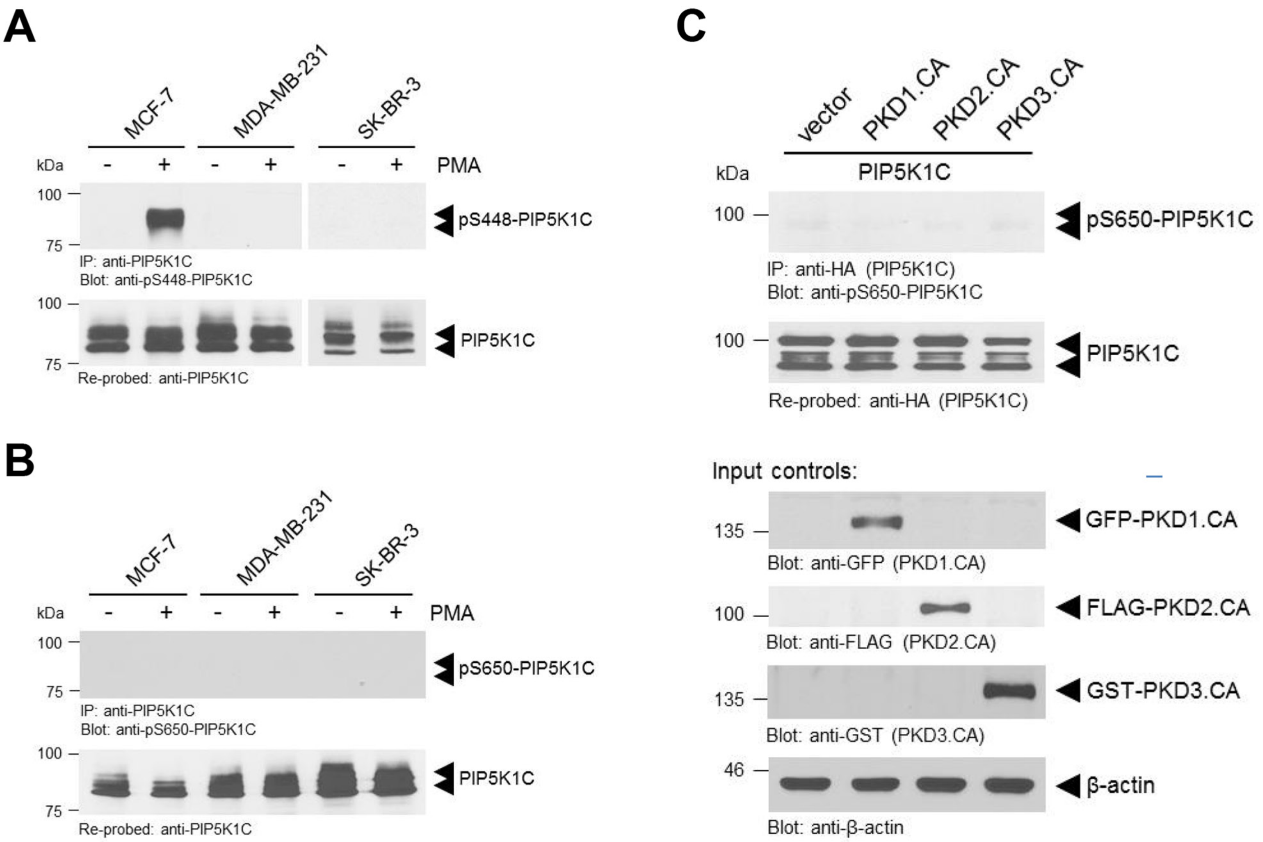

Supplementary Figure 1: PIP5K1C phosphorylation at S448 and S650 in breast cancer cell lines. (A, B) Indicated cell lines were treated with DMSO control or PMA (100 nM) for 10 min. Cells were lysed, endogenous PIP5K1C was immunoprecipitated (anti-PIP5K1C), and immunoprecipitates were analyzed by SDS-PAGE and immunoblotting for phosphorylation of PIP5K1C at S448 (anti-pS448-PIP5K1C, A) or at S650 (anti-pS650-PIP5K1C, B). Samples were re-probed for total PIP5K1C. **(C)** MCF-7 cells were transfected with tagged constitutively-active versions of PKD1, PKD2 or PKD3 together with HA-tagged PIP5K1C. Cells were lysed, overexpressed PIP5K1C was immunoprecipitated (anti-HA), and immunoprecipitates were analyzed by SDS-PAGE and immunoblotting for phosphorylation of PIP5K1C at S650 (anti-pS650-PIP5K1C). Samples were re-probed for total PIP5K1C by staining with anti-HA. In addition expression of active PKD isoforms was determined by Western blotting of lysates with TAG-specific antibodies as indicated.

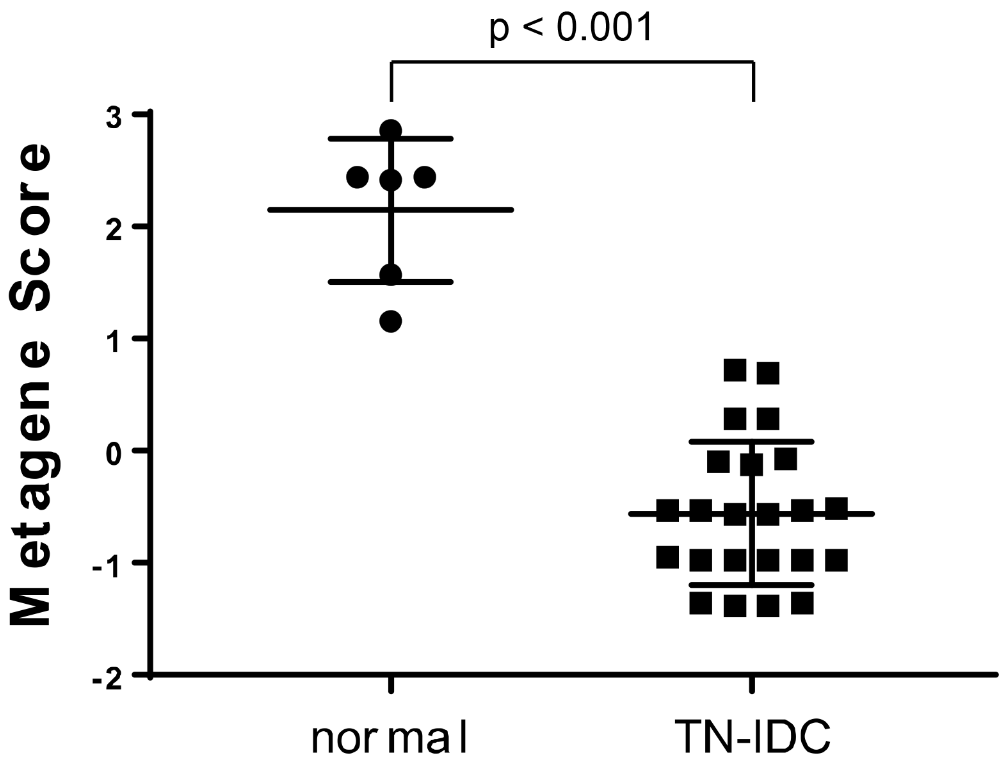

Supplementary Figure 2: Statistical significance between normal and TN-IDC samples in Figure 6B. The metagene score in this figure is the first principle component determined by cluster analysis of IHC scores for PKD1 and pS448-PIP5K1C for all samples.
